# Domestication‐induced reduction in eye size revealed in multiple common garden experiments: The case of Atlantic salmon (*Salmo salar* L.)

**DOI:** 10.1111/eva.13297

**Published:** 2021-09-21

**Authors:** William Bernard Perry, Joshka Kaufmann, Monica Favnebøe Solberg, Christopher Brodie, Angela Maria Coral Medina, Kirthana Pillay, Anna Egerton, Alison Harvey, Karl P. Phillips, Jamie Coughlan, Fintan Egan, Ronan Grealis, Steve Hutton, Floriane Leseur, Sarah Ryan, Russell Poole, Ger Rogan, Elizabeth Ryder, Patrick Schaal, Catherine Waters, Robert Wynne, Martin Taylor, Paulo Prodöhl, Simon Creer, Martin Llewellyn, Philip McGinnity, Gary Carvalho, Kevin Alan Glover

**Affiliations:** ^1^ Molecular Ecology and Fisheries Genetics Laboratory School of Biological Science Bangor University Bangor, Gwynedd UK; ^2^ Water Research Institute School of Biosciences Cardiff University Cardiff UK; ^3^ Population Genetics Research Group Institute of Marine Research Bergen Norway; ^4^ School of Biological, Earth and Environmental Sciences University College Cork Cork Ireland; ^5^ Marine Institute Furnace, Newport Co. Mayo Ireland; ^6^ Ecosystems and Environment Research Centre School of Environment and Life Sciences University of Salford Salford UK; ^7^ School of Microbiology Food Science & Technology Building University College Cork Cork Ireland; ^8^ Institute of Biodiversity Animal Health & Comparative Medicine University of Glasgow Glasgow UK; ^9^ School of Biological Sciences University of East Anglia Norwich UK; ^10^ Institute for Global Food Security School of Biological Sciences Medical Biology Centre Queen’s University Belfast UK; ^11^ Institute of Biology University of Bergen Bergen Norway

**Keywords:** allometry, aquaculture, domestication, escapees, introgression, morphology

## Abstract

Domestication leads to changes in traits that are under directional selection in breeding programmes, though unintentional changes in nonproduction traits can also arise. In offspring of escaping fish and any hybrid progeny, such unintentionally altered traits may reduce fitness in the wild. Atlantic salmon breeding programmes were established in the early 1970s, resulting in genetic changes in multiple traits. However, the impact of domestication on eye size has not been studied. We measured body size corrected eye size in 4000 salmon from six common garden experiments conducted under artificial and natural conditions, in freshwater and saltwater environments, in two countries. Within these common gardens, offspring of domesticated and wild parents were crossed to produce 11 strains, with varying genetic backgrounds (wild, domesticated, F1 hybrids, F2 hybrids and backcrosses). Size‐adjusted eye size was influenced by both genetic and environmental factors. Domesticated fish reared under artificial conditions had smaller adjusted eye size when compared to wild fish reared under identical conditions, in both the freshwater and marine environments, and in both Irish and Norwegian experiments. However, in parr that had been introduced into a river environment shortly after hatching and sampled at the end of their first summer, differences in adjusted eye size observed among genetic groups were of a reduced magnitude and were nonsignificant in 2‐year‐old sea migrating smolts sampled in the river immediately prior to sea entry. Collectively, our findings could suggest that where natural selection is present, individuals with reduced eye size are maladapted and consequently have reduced fitness, building on our understanding of the mechanisms that underlie a well‐documented reduction in the fitness of the progeny of domesticated salmon, including hybrid progeny, in the wild.

## 1 INTRODUCTION

The eye, a structure used for photoreception and vision, shows great diversity in its complexity throughout the animal kingdom, including light‐sensing cells (e.g. annelids like leeches), pinhole eyes (e.g. molluscs like chambered nautili) and anatomically complex structures seen in vertebrates (Schwab, 2018). Many vertebrate taxa depend on visual stimuli, alongside other sensory inputs to undertake basic functions such as food detection, predator avoidance, and interacting, and mating, with conspecifics. Adaptations in eye morphology facilitate better collection of visual stimuli; for example, among visual‐feeding reef fish, eye size is larger in nocturnal species in order to achieve better light sensitivity (Schmitz & Wainwright, 2011). Similarly, larger eye size is also seen in nocturnal primates (Kirk, 2006). However, unlike primates, most teleost fish have pupils that lack significant mobility and thus have a pupil that is fixed in size (Douglas, 2018). Other than increasing sensitivity to light, larger eyes also provide visual acuity (the ability to resolve spatial detail) (Caves et al., 2017), which has driven larger eye sizes in freshwater fish, such as common galaxias (*Galaxias maculatus* Jenyns, 1842) in locations where they actively feed on zooplankton (Mercer et al., 2020).

In addition to feeding, visual cues can be important in navigating an environment, especially in highly mobile species. For example, the synchronized diel vertical migration of marine fish, the largest known daily migration among animals (Brierley, 2014), is driven in part by visual cues (Häfker et al., 2017). Vision is also involved in a large component of predator–prey interactions in fish, and it has been suggested that the arms race resulting from the evolution of good visual systems fuelled the evolution of complex animals (Barbosa & Castellanos, 2005). The enlarged eye of giant squid (*Architeuthis* sp), which has been measured at 27 cm, is thought to be driven by the need for enhanced light sensitivity in a light‐scarce environment in order to avoid predation (Nilsson et al., 2012). Experimental evidence of predation increasing eye and pupil size in prey through adaptive plastic responses has been demonstrated multiple times in fish, such as male fathead minnows (*Pimephales promelas*) (Meuthen et al., 2019), three‐spined sticklebacks (*Gasterosteus aculeatus*) (Ab Ghani et al., 2016) and crucian carp (*Carassius carassius*) (Vinterstare et al., 2020). Therefore, there is potential for the traits relating to vision, such as eye size, to be under strong natural selection, and to become genetically fixed within a population or species.

Despite the benefits of large, complex eyes, they come at a cost. One of the most fundamental costs is the associated metabolic expenditure (Huang et al., 2018). A comprehensive example demonstrating metabolic expenditure of eyes is that of the eyeless Mexican cavefish (*Astyanax mexicanus* De Filippi, 1853). By examining *A*. *mexicanus* ecotypes that have not lost their vision, it has been calculated that the eye and corresponding neural tissue can account for up to 15% of the fish’s resting metabolism energy requirements (Moran et al., 2015). Examples have also shown that large eyes can increase the risk of predation, as shown in Eurasian perch (*Perca fluviatilis*), that are at a higher risk of predation due to the reflective layers behind the eye, making them more conspicuous to predators (Svanbäck & Johansson, 2019).

Teleost fish provide excellent study organisms for examining the influence of ecology and evolution on eye form and function. One such iconic teleost fish species that has a diverse life history in terms of habitat (rivers, lakes and sea) and ontogeny (alevins, fry, parr, smolt, postsmolt, adults, mature parr) and that relies heavily on visual cues, is the Atlantic salmon (*Salmo salar* L.). Although the species is not an obligate visual feeder, Atlantic salmon utilize visual cues to feed (Jonsson et al., 2017). Studies on juvenile Atlantic salmon have shown that feeding rates are 7.5 times higher during daylight hours than at night in a hatchery setting (12‐hr light, 12‐hr dark) (Jørgensen & Jobling, 1992). The same trend has also been seen in studies examining the effect of natural night light intensities on feeding in juvenile Atlantic salmon, including in hatchery settings, where feeding efficiency at the highest natural illumination levels was only 35% of the efficiency seen during daylight feeding (Fraser & Metcalfe, 1997). There is evidence of fine‐scale local adaptation in eye size in Atlantic salmon and brown trout systems. In Ireland, both Atlantic salmon and brown trout (*Salmo trutta*) parr in the Burrishoole catchment have been found to have larger eyes than fish found in the Barrow catchment. Larger eye size in the Burrishoole catchment is likely due to the lower visibility, peat‐stained, waters and has been hypothesized to be an adaptation to locating and ingesting prey in low visibility conditions (Drinan et al., 2012). There is also evidence to show that visual cues could play a role in the migration of salmon back to their natal spawning grounds, with blind sockeye salmon taking longer to complete their journey (Ueda et al., 1998). The reduction in fitness due to loss of visual cues is also highlighted by the reduced growth performance caused by cataract formation during smoltification in farm environments (Sveier et al., 2001). Visual stimulus via photoperiod also has an important role in the physiological changes associated with smoltification (McCormick et al., 1998), as well as migration both up and downstream (Jonsson, 1991).

Many aquatic species in recent decades have experienced large changes due to domestication (Teletchea, 2019). Domestication is a multigenerational process in which humans seek to adapt an organism to human needs, usually increased production in a specific controlled environment through captive rearing and artificial selection. The recent domestication of Atlantic salmon in a farmed setting provides a ~13–15 generation long experiment to identify the consequences of relaxed natural selection and exposure to artificial selection. Large‐scale Atlantic salmon aquaculture commenced in the early 1970s, and the relatively recent introduction of modern aquaculture techniques has facilitated the rapid growth of salmon farming into a massive commodity industry (Kumar et al., 2018). Artificial selection has successfully altered traits such as body weight, age of sexual maturation, flesh colour and fat content in domesticated strains. Strong directional selection, along with exposure to artificial environments, has also resulted in the divergence of many nonproduction traits from wild populations (reviewed in Glover et al., 2017).

There are many examples of divergent phenotypes between wild and domesticated salmon. Some examples include domesticated fish having a reduced maximum rate of oxygen uptake (Zhang et al., 2016), higher levels of aggressive behaviour (Einum & Fleming, 1997) and changes to external morphology (Fleming & Einum, 1997; Perry et al., 2019; Pulcini et al., 2013; Wessel et al., 2006). Consequently, combined genetic, morphological and behavioural divergence seen in domesticated Atlantic salmon is likely to result in reduced lifetime reproductive success of domesticated progeny in the wild (Besnier et al., 2015; Fleming et al., 2000; McGinnity et al., 1997, 2003; Skaala et al., 2019; Solberg et al., 2020).

Common garden studies are useful in identifying the relative roles of environmental and genetic control over traits that change during domestication. However, such studies only examine differences within specific environments, and so trends seen in hatchery‐based common garden studies are not necessarily directly transferable to other environments. To gain a representative ecological perspective of domestication‐induced morphological changes, a reciprocal common garden experiment provides a powerful tool to investigate selection across differing environments (De Villemereuil et al., 2016). Such a study, involving multiple common garden experiments to disentangle the role of genetic and environmental factors on eye size has to date not been conducted in Atlantic salmon. Common garden experiments investigating the relative performance of progeny from domesticated, hybrid and wild salmon have been undertaken at the Institute of Marine Research (IMR), Norway, for more than a decade (e.g. Bicskei et al., 2016; Glover, Harvey, et al., 2020; Glover, Wennevik, et al., 2020; Harvey, Solberg, Glover, et al., 2016; Solberg, Skaala, Nilsen, Glover, 2013; Solberg, Zhang, Nilsen, Glover, 2013). The process of rearing fish in these common garden experiments involves a large pedigree‐based population of salmon consisting of fish originating from wild and domesticated genetic backgrounds, including respective hybrids and backcrosses. Similarly in Ireland, the Marine Institute facility on the Srahrevagh River in the Burrishoole catchment in the west of Ireland has been the location for a series of common garden experiments undertaken in the wild since the early 1990s. In these studies, the relative fitness of the offspring of wild native, non‐native, captive bred native and domesticated salmon (de Eyto et al., 2011; McGinnity et al., 1997, 2003; O’Toole et al., 2015).

**FIGURE 1:**
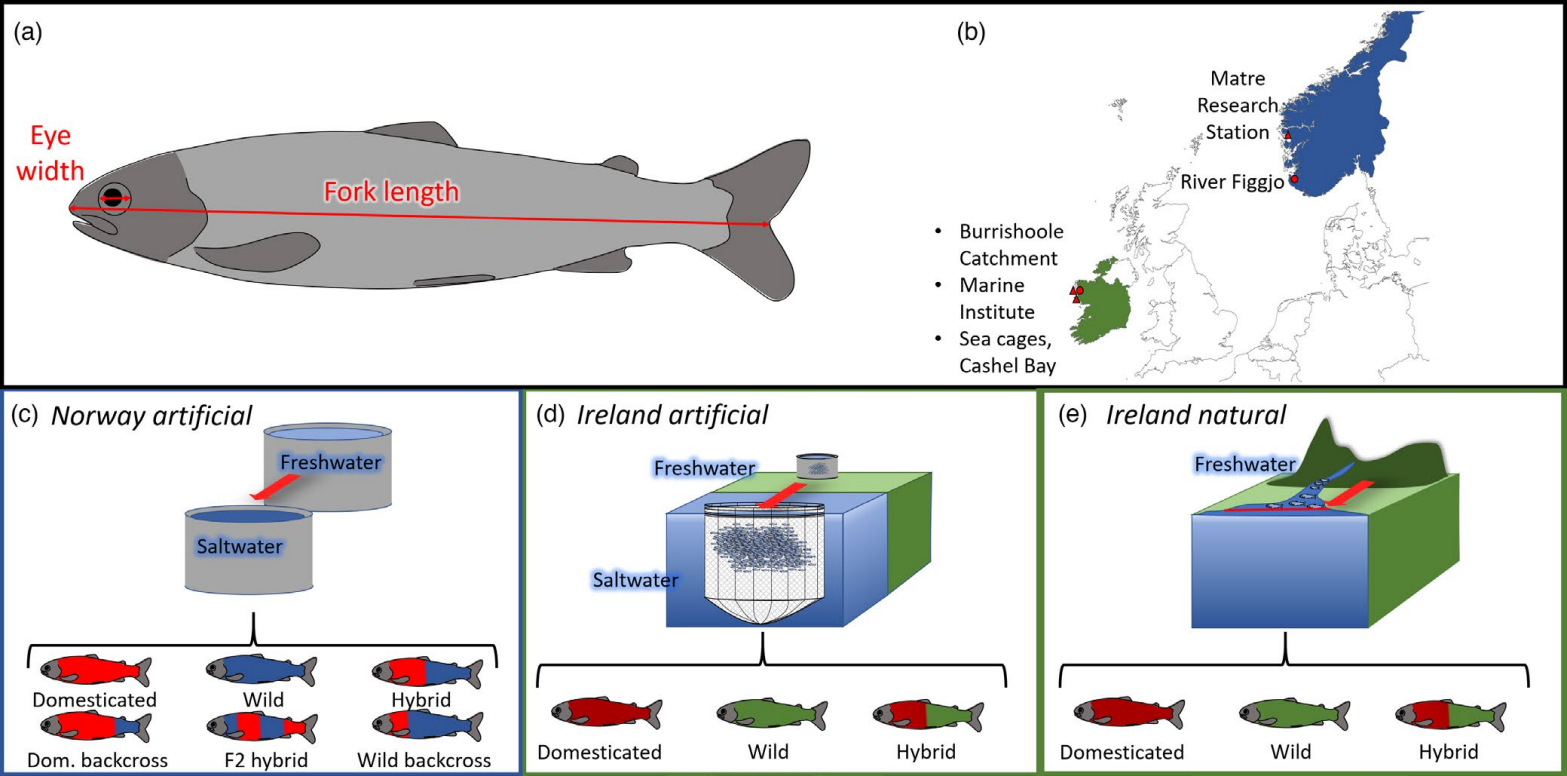
Diagram showing (a) eye width (henceforth, eye size) and fork length. In addition to the breakdown of experimental designs used in this study between the origins of (b) Norway (blue) and Ireland (green), including the sites where the wild genetic backgrounds were acquired. What is also shown is the three different experiment types, including (c) Norwegian fish reared under artificial conditions, (d) Irish fish reared under artificial conditions and (e) Irish fish reared under natural conditions, with corresponding genetic backgrounds. Wild and domesticated genetic backgrounds each have a unique colour, with the mixing of those colours demonstrating hybridization between genetic backgrounds. It should be highlighted that Irish fish reared under natural conditions (e) in the saltwater life stage are captured before they enter the marine environment

Here, we present the most comprehensive analysis to date on Atlantic salmon, to examine the effects of domestication on body length‐adjusted eye size, comprising of ~4000 offspring sampled from several common garden experiments undertaken in contrasting natural and artificial environments, including freshwater (parr and sea migrating smolt life stages) and marine environments (postsmolt life stage), originating from 11 genetic backgrounds (Figure 1), replicated in both Ireland and Norway. Based on previous meta‐analyses examining domestication‐induced morphological change in fish (Wringe et al., 2016), we predict that the progeny of domesticated parents would have reduced relative eye size.

## 2 MATERIALS AND METHODS

### 2.1 Common garden experiments in Norway

Eye and body length measurements were obtained from an experimental population that was established in 2015 at the Matre Research Station, Masfjorden, Norway (60°52′26.4″N, 5°35′09.0″E), using domesticated (Mowi) and wild (Figgjo) parents and subsequently reared under artificial, tank‐based, freshwater and saltwater environments. The production of F1 fish followed the protocol and design of Solberg et al. (2014) and resulted in a study population of seven groups of varying genetic background: domesticated (Norwegian Mowi—6 families); wild (from the river Figgjo, Norway—6 families); hybrid domesticated female and a wild male (HFF—3 families); hybrid wild female and a domesticated male (HWF—3 families); F2 hybrid (6 families); wild backcross (6 families); domesticated backcross (6 families) (Figure 1c), with all groups being mixed at the eyed egg stage. During the freshwater life stage, fish were reared in a flow through system of four replicated tanks, located in an enclosed outbuilding. The rearing tanks were 3 m wide, 1.25 m deep, 6300 L in volume and were fed from a continuous flow source at 60 L/min, with incoming water passing through a 40‐μm filtration unit before entering individual tanks. Photographs were taken in April 2017 of 1116 individuals prior to the transfer of a subset of the experimental population from freshwater to saltwater tanks, and tissue was stored in 100% ethanol for subsequent parentage assignment. The saltwater tanks were also based on a flow through system with water taken from the adjacent fjord with a flow rate of 170–200 L/min. The tanks were 5 m wide, 1.1 m deep and 15,600 L in volume. Both freshwater and saltwater tanks were artificially lit, starting with a 24‐h light regime during first feeding, with the photoperiod simulating that of Bergen post first feeding. Fish were fed on a diet of pellets produced by Skretting Nutra Olympic (Cheshire, UK). Photographs were taken in April 2018 of 784 saltwater reared postsmolts, and tissue was again secured for subsequent parentage assignment. The common garden design implemented here makes comparisons between freshwater and saltwater life stages particularly robust as we were comparing siblings (e.g. domesticated freshwater compared to their siblings in saltwater). As the fish were kept under standard rearing conditions and no procedures were carried out, no specific research permit was required. All those working directly with the experimental animals had undergone Norwegian Food Safety Authority (NFSA) training.

### 2.2 Common garden experiments in Ireland

In Ireland, eye size data were obtained for the progeny of domesticated and wild parents from two experimental populations established in December 2016 and 2017 and reared as part of common garden experiments in farm and natural river environments at the Marine Institute facility at Furnace, Newport, Co. Mayo (53°55′22″N 9°34′18″W). The Irish experiments included the offspring of domesticated (derived from Norwegian Mowi farmed in Ireland since the mid‐1980s), wild (from the Burrishoole River system), domesticated female and wild male hybrids (HFF) and wild female and domesticated male hybrids (HWF) (Figure 1d,e).

From the December 2016 cohort, data were obtained from the photographs of 67 sea migrating smolts sampled in April and May 2019 immediately prior to sea entry (Figure 1e). These fish had been introduced into the Srahrevagh River, a headwater tributary of the Burrishoole River, as swim‐up fry (shortly after hatching on the onset of exogenous feeding) 2 years previously as part of a common garden experiment and were therefore subject to natural selection. The experimental section of the Srahrevagh river is comprised of approximately 7250 m^2^ of natural juvenile salmonid habitat contained at its lower end by a fish trap capable of capturing all life‐history stages (fry to adult), and at its upper end by a series of large waterfalls. The Srahrevagh river is a third‐order upland stream with a medium to high gradient (discharge = 25,200 L/min). This river has variable, but usually very high colour (median colour concentration of 130 mg PtCo/L) due to high dissolved organic carbon from the oxidation of peaty soils during dry weather (Doyle et al., 2019). Juvenile salmon feed actively on adult Diptera, Plecoptera and Trichoptera, as well as Coleoptera (de Eyto et al., 2020). For further details on the Srahrevagh river, see McGinnity et al. (1997).

Eye and body size data were available for the December 2017 cohort fish reared and sampled in three different environments. As had been done for the 2016 experimental population, swim‐up fry from the 2017 cohort were introduced into the Srahrevagh River in March 2018. Photographs for eye and body size analyses were taken from a sample of 756 summer parr collected from the river in October 2018 (Figure 1e).

In October 2018, 778 individuals, in addition to tissue samples for genetic analysis, were photographed from a sample of tank‐reared 2017 cohort parr obtained from the freshwater hatchery‐based common garden experiment (Figure 1d). These fish had been reared since first feeding in four flow through outdoor tanks fitted with nets to exclude avian and mammalian predators. Tanks were 2.5 m wide, 0.6 m deep and 2400 L volume, fed by a continuous flow of freshwater 60 L/min from Lough Feeagh, a freshwater lake located upstream from the hatchery. The intake pipes in Lough Feeagh are screened to prevent large debris entering the system, but water is otherwise unfiltered. Water taken into the tanks from the lake can contain high levels of suspended solids and colour that darken the water, particularly after heavy rain. Freshwater fish reared in the hatchery were fed *ad libitum* on a diet of Skretting Nutra Olympic pellets.

In April 2018, a subset of the 2017 cohort was transferred to the Marine Institute's sea farm (Lehanagh Pool, Breartrach Bui, Co.; 53°21′11.7″N 9°55′42.7″W) (Figure 1d). Subsequent to this transfer, a third sample of 213 postsmolts was photographed from fish collected from the sea cages between June and August 2018. The fish in the sea cage were fed daily on a diet of Ewos 75 pellets produced by Cargill.

Genetic parentage assignments were based on microsatellite genotypes, obtained from tissue samples collected at the time of sampling. The protocols used in the genetic analyses are provided in the Supplementary materials. The Irish studies were carried out under a Health Products Regulatory Authority (HPRA) licence number AE19130‐P056.

### 2.3 Photograph preparation and data collection

After fork length of the fish had been measured (to the nearest 1 mm) on a standard measuring board, photographs were taken on a digital single‐lens reflex camera placed above samples on a level surface, with a scale in shot under natural light. Fish collected from the river experiment were measured using callipers to the nearest 0.01 mm. Before the addition of landmarks, all photographs were quality checked, without knowledge of genetic background and low‐quality images (when landmarks could not be applied) removed. Eye width, hereon in referred to as eye size, was measured from these photographs using tpsDig v 2.28 (Rohlf, 2016) by one person. Fork length measured during sampling and body length estimated from the photographs was used to scale the linear measurements obtained from the landmark data. After quality control, a total of 3970 unique photographs were used in subsequent analyses.

### 2.4 Fork length—statistical analysis

A log10 transformation of fork length was used as response variable in two linear mixed‐effect models, (LMMs), performed separately for Norwegian and Irish experiments. The division of the dataset was necessary to allow for a full factorial design within each model, as family and tank replicate information was not available for Irish saltwater postsmolts. As a means of uncovering variation explained by the independent variables, including random effects, LMMs were chosen to analyse the data. All LMMs were constructed using the R package “lme4” (Bates et al., 2015). The model included as fixed factors: genetic background and life stage (freshwater and saltwater—in the natural common garden experiment, “freshwater” and “saltwater,” respectively, refer to freshwater parr and smolts leaving the river to go to sea), full interaction terms between factors, as well as the random factor of “sampling date.” The LMM constructed for the Irish experiment also included the fixed factor “rearing,” for artificially and naturally reared fish. The random factors “family” and “tank replicate” were included in the model assessing fork length in the Norwegian artificially reared fish. Estimated marginal means and pairwise comparisons between means were calculated using the R package “emmeans” (Lenth et al., 2018).

### 2.5 Eye size—statistical analysis

Digital eye measurements were scaled by using the scale between the body lengths obtained from the photographs and fork lengths, which were measured at the same time as sampling. Once eye size had been scaled, the dataset was split by experiment (e.g. Norwegian artificially reared, Irish artificially reared, Irish naturally reared). We analysed the Irish and Norwegian datasets separately, as preliminary analyses suggested the two experiments had different allometry coefficients (Figure S1). Moreover, splitting the dataset also allowed the inclusion of experimental specific variables (family and tank), as mentioned previously. After being split, three separate linear regressions were constructed for Norwegian artificially reared (*R* ^2^ = 0.91, *p* < 0.01), Irish artificially reared (*R* ^2^ = 0.78, *p* < 0.01), Irish naturally reared fish (*R* ^2^ = 0.86, *p* < 0.01). A fork length‐adjusted measure for eye size was calculated using the residuals from these three log‐log regressions between fork length and eye size. Body size‐adjusted eye size is henceforth referred to as “adjusted eye size.” Adjusting for body size using regressions is a method that has been shown previously to be more statistically robust than simple divisional indices (Perry et al., 2020). The residuals were then used as response variables in three different LMMs using “lme4” (Bates et al., 2015). The LMMs included the fixed factors: genetic background (wild, domesticated, HWF and HFF) and life stage (freshwater and saltwater), full interaction terms between factors, as well as the random factor of “sampling date.” “Family” and “tank” were also included as random factors in the case of the Norwegian artificially reared fish, but “tank” was removed from the model using a step function. The “step” function within “lme4” was used to select the best fitting model through automatic backward elimination, removing fixed terms and random factors that did not significantly improve the model. Norwegian artificially reared fish also had additional levels for genetic background: wild backcross, domesticated backcross and F2 hybrid. Estimated marginal means and pairwise comparisons between means were calculated using “emmeans” (Lenth et al., 2018). To compare the variance explained by the fixed effects across the different experiments, we extracted semi‐partial *R* ^2^ values using r2glmm::r2beta (Jaeger et al., 2017). The semi‐partial (marginal) *R* ^2^ describes the variance explained by each fixed effect adjusted for the other predictors in the mixed models on relative eye size. Those were extracted from the selected models and not adjusted by the general/total variance explained by each model.

All statistical analyses were carried out in R v. 3.6.2 (R Core Team, 2017), with data and code available in the electronic Supplementary material.

## 3 RESULTS

### 3.1 Fork length—Norwegian fish

Means and standard error for each genetic background in the different experimental groups can be found in Table S1. Here, we describe the key length patterns.

**FIGURE 2:**
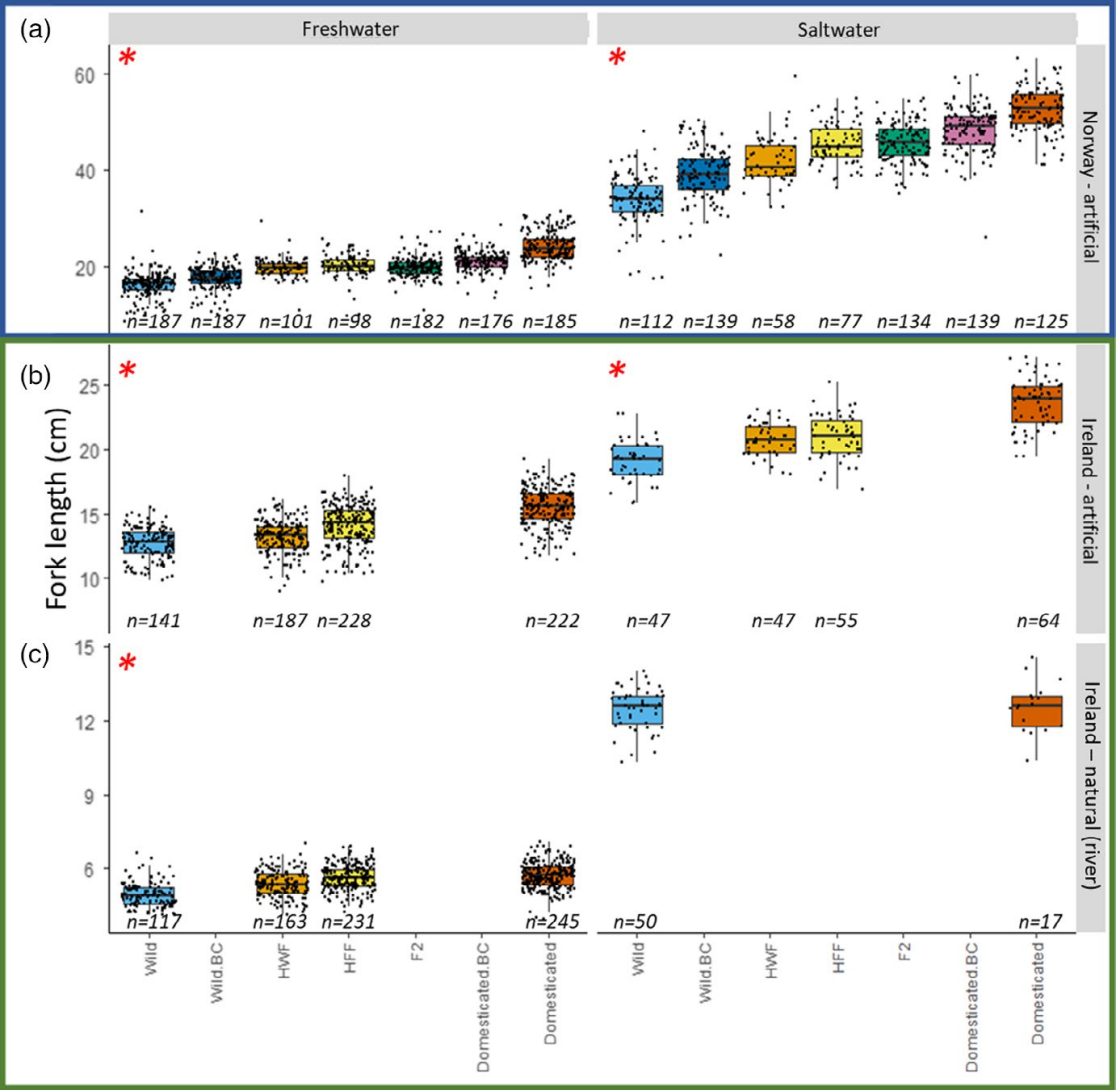
Fork length broken down by genetic background and life stage in the (a) Norwegian artificially reared fish, (b) Irish fish reared under artificial conditions and (c) Irish fish reared under natural (river) conditions, along with their corresponding samples sizes. It should be highlighted that the *y*‐axis shows different scales in order to allow for trends within smaller fish. In addition to this, it should also be highlighted that Irish fish reared under natural (river) conditions (c) are captured as migrating smolts in the river before they enter the marine environment. Red asterisk corresponds to a significant effect of genetic background

There was a significant interaction term between genetic background and life stage (*F*_6,1851_ = 8.12, Sum Sq = 0.122, *p* < 0.001), meaning that the effect of genetic background upon log10‐transformed fork length (henceforth, length) differed between the two life stages (freshwater and saltwater). There was a significant effect of genetic background on length in the Norwegian artificially reared fish (*F*_6,29_ = 34.27, Sum Sq = 0.516, *p* < 0.001) (Figure 2a). Norwegian freshwater domesticated fish were significantly longer (mean = 1.37 ± 0.013 [SE]) than Norwegian freshwater wild fish (mean = 1.19 ± 0.013; mean difference of 7.96 cm, *t*_32_ = 11.88, *p* < 0.001). Norwegian saltwater domesticated postsmolts were also significantly larger (mean = 1.71 ± 0.013) than Norwegian saltwater wild postsmolts (mean = 1.52 ± 0.013) (mean difference of 18.17 cm, *t*_37_ = 12.68, *p* < 0.001).

### 3.2 Fork length—Irish fish

With length, all interaction terms between factors were significant, including genetic background and life stage (*F*_3,836_ = 7.75, Sum Sq = 0.042, *p* < 0.001), genetic background and rearing conditions (*F*_3,446_ = 27.10, Sum Sq = 0.148, *p* < 0.001), life stage and rearing (*F*_1,20_ = 377.11, Sum Sq = 0.684, *p* < 0.001). There was also a three‐way interaction between genetic background, life stage and rearing (*F*_1,769_ = 12.64, Sum Sq = 0.023, *p* < 0.001), implying that the effect of genetic background upon length differs among life stages and rearing type. There was a significant effect of genetic background on length in the Irish fish (*F*_3,1219_ = 82.63, Sum Sq = 0.450, *p* < 0.001) (Figure 2b). Reared under artificial conditions, Irish domesticated fish were significantly larger than wild fish as both freshwater parr (dom. = 1.19 ± 0.005; wild = 1.10 ± 0.005; *t*_142_ = 15.22, *p* < 0.001) and saltwater postsmolts (dom. = 1.37 ± 0.006; wild = 1.28 ± 0.006; difference of 4.39 cm, *t*_1758_ = 11.05, *p* < 0.001).

Irish freshwater domesticated parr reared under natural conditions were significantly longer (mean = 0.755 ± 0.0049) than wild parr (mean = 0.691 ± 0.0057; difference of 0.78 cm, *t*_1798_ = 13.16, *p* < 0.001). However, no significant pairwise differences were seen between the migrating smolts from domesticated and wild parents reared under natural conditions in the Irish river (*t*_1384_ = 0.26, *p* > 0.99).

### 3.3 Eye size—Norwegian fish, artificial conditions

**FIGURE 3:**
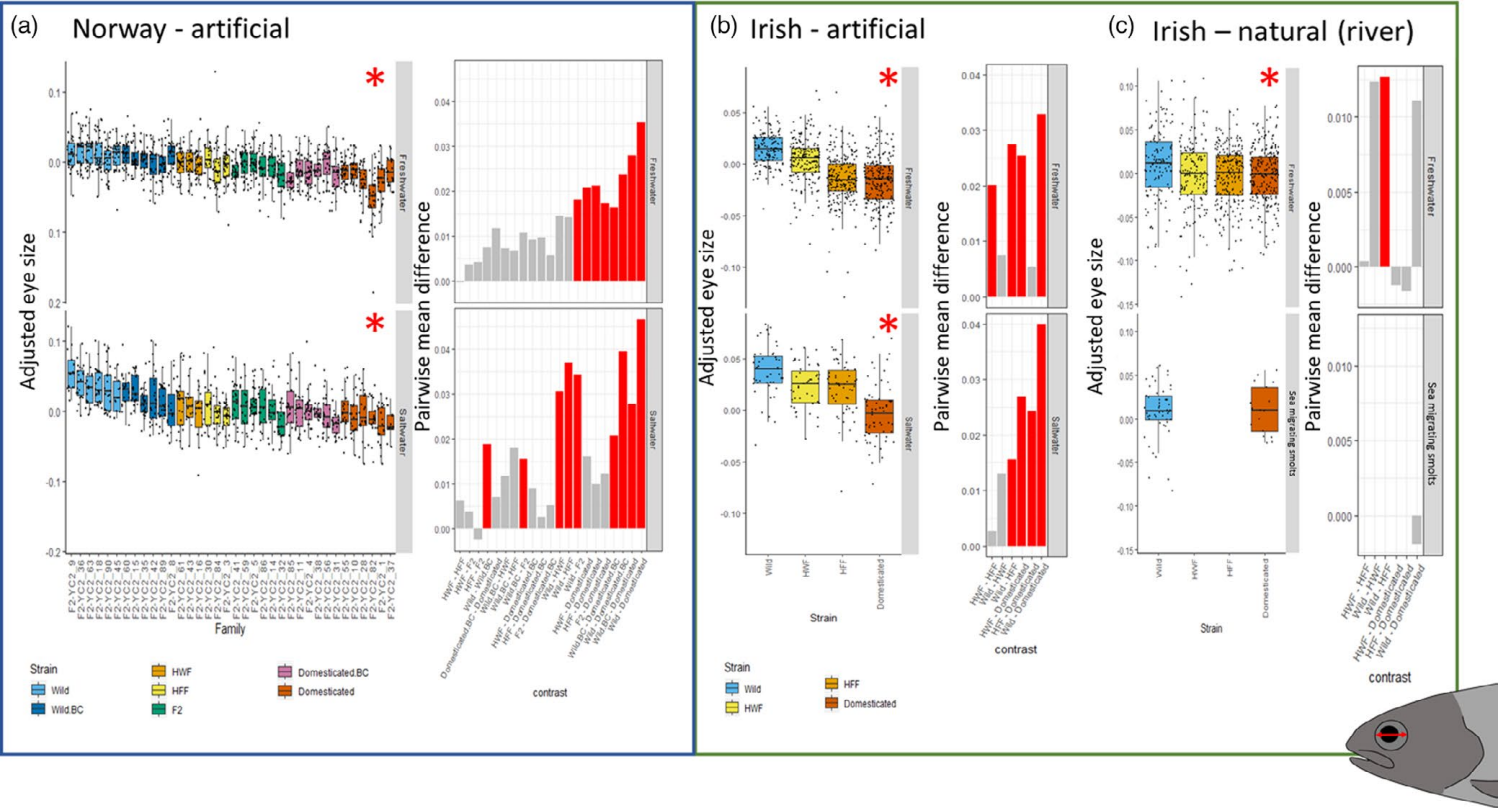
Fork length adjusted eye size between genetic backgrounds (wild, hybrid farmed female (HFF), hybrid wild female (HWF) and domesticated) split into (a) Norwegian artificially reared (where family information is included), (b) Irish fish reared under artificial conditions and (c) Irish fish reared under natural (river) conditions. It should also be highlighted that Irish fish reared under natural (river) conditions (c) are captured as migrating smolts in the river before they enter the marine environment. Red asterisk corresponds to significant pairwise differences between genetic backgrounds. Pairwise differences of mean adjusted eye size between genetic backgrounds are also included, produced from the LMMs using emmeans, and significant differences in means are coloured red, while grey represent nonsignificant differences

Genetic background (*F*_6,30_ = 23.08, Sum Sq = 0.08, *p* < 0.001) and the interaction between genetic background and life stage (*F*_6,1853_ = 5.09, Sum Sq = 0.02, *p* < 0.001) had a significant effect on adjusted eye size in Norwegian artificially reared fish, implying that the effect of genetic background upon adjusted eye size differed between freshwater and saltwater life stages. There were many significant pairwise differences between genetic backgrounds (Figure 3a); however, the overall trend in both the freshwater and saltwater life stages was for larger adjusted eye size in wild individuals compared to domesticated individuals, with hybrids and backcrosses showing intermediate phenotypes.

### 3.4 Eye size—Irish fish, artificial conditions

Genetic background (*F*_3,976_ = 75.41, Sum Sq = 0.10, *p* < 0.001) and life stage (*F*_1,22_ = 23.6, Sum Sq = 0.01, *p* < 0.001) had a significant effect on adjusted eye size in Irish artificially reared fish, with a significant interaction between genetic background and life stage (*F*_3,976_ = 5.72, Sum Sq = 0.01, *p* < 0.001), demonstrating that the effect of genetic background upon adjusted eye size deviated between the two life stages. There were many significant pairwise differences between genetic backgrounds (Figure 3b); however, the overall trend in both the freshwater and saltwater life stages was larger adjusted eye size in wild individuals compared with domesticated individuals, with hybrids and backcrosses showing intermediate phenotypes (Figure 3b).

### 3.5 Eye size—Irish fish, natural conditions

We found no significant effect of genetic background on adjusted eye size in fish reared under natural conditions (*F*_3,796_ = 2.43, Sum Sq = 0.009, *p* = 0.065; Figure 3c), and no significant effect of life stage (LME life stage: *F*_1,30_ = 0.71, Sum Sq = 0.001, *p* = 0.41) or the interaction term between genetic background and life stage (*F*_1,718_ = 1.45, Sum Sq = 0.002, *p* = 0.23) (Figure 3c). The effect of genetic background on adjusted eye size under natural conditions, albeit not statistically significant, nevertheless mirrored the overall direction seen in all other experiments with wild fish having larger adjusted eye size than domesticated fish. This was partly supported by a significant pairwise comparison between wild and HFF (*t*_816_ = 3.14, *p* = 0.04).

Semi‐partial *R* ^2^ values demonstrated that genetic background and life stage both explained a larger proportion of variance in eye size under artificial conditions than under natural conditions (Figure S2).

## 4 DISCUSSION

Using data from multiple common garden experiments carried out under natural river, hatchery tank and sea farm conditions, we demonstrate that Atlantic salmon eye size is influenced by both genetic background and environment. Under artificial rearing conditions, the progeny of domesticated (farmed) fish had smaller adjusted eye size when compared to the progeny of wild fish in both the freshwater and marine environments. This was consistent across farm‐based experiments conducted in both Ireland and Norway. In contrast, differences in adjusted eye size were less apparent among genetic groups when reared under natural river conditions, potentially due to reduced survival of individuals with reduced relative eye size in situations where there is natural selection. Therefore, domestication‐mediated changes in eye size as found here could be an important mechanism contributing to previously well‐documented reductions in the fitness of the offspring of domesticated salmon in the wild.

### 4.1 Reduced adjusted eye size

Artificially reared domesticated fish had significantly smaller adjusted eye size than artificially reared wild fish, with hybrids showing an intermediate phenotype (Figure 3), suggesting an additive genetic basis to the trait. Indeed, genetic background explained more variation in eye size as contrasting life stages (Figure S2). Smaller adjusted eye size associated with domestication has also been observed in coho salmon (*Oncorhynchus kisutch*) and rainbow trout (*Oncorhynchus mykiss*) that have undergone growth hormone (GH) transgenesis. Growth hormone transgenesis in these species reduced levels of insulin‐like growth factor I mRNA in the eye when compared to other organs, thus suggesting that eye growth is regulated differently from total growth (Devlin et al., 2012). Therefore, it is possible that selection for overall total body growth in farmed Atlantic salmon has been selecting only for pathways that increase overall growth, and not for growth pathways that increase the size of specific organs such as the eye. Indeed, separate pathways for somatic growth and neurological growth have shown similar trends in other domesticated species, which is pertinent as the eyes are a sensory extension to the central nervous system, with shared developmental origins (Casarosa et al., 1997). These differing pathways for somatic and neurological growth have previously been used to refute the notion that domestication reduces relative brain size, demonstrating that the brain can remain the same size, but due to the larger body size of domesticated individuals, allometric scaling makes it appear that brain size has decreased (Henriksen et al., 2016). However, many studies have also suggested that reduction in the size of the brain observed in fish when they are reared in different artificial environments, where allometric scaling has been controlled for, is due to phenotypic plasticity. For example, rainbow trout alevins have shown a significantly reduced cerebellum size in artificial environments (Kihslinger & Nevitt, 2006). In addition, Atlantic cod (*Gadus morhua*) and Atlantic salmon alevins also show a significant reduction in brain size when compared to fish reared in more complex environments (Mayer et al., 2011; Näslund et al., 2012). Although interpreted by the authors of these studies as a plastic response, it provides good evidence that there is selection for the reduction of organ sizes in artificial environments, such as the brain, and by extension, the eye, irrespective of allometry.

The reduced adjusted eye size seen in our study could also be due to pleiotropic effects linked with artificial selection. The fundamental reason why domesticated salmon grow larger than wild fish under artificial conditions when fed *ad lib* rations is not yet fully understood. Genetically increased appetite has been suggested in Atlantic salmon (Harvey, Solberg, Troianou, et al., 2016). Indeed, experimental evidence has shown that overexpression of growth hormone in transgenic coho salmon modulates the effect of peptides involved in appetite suppression (White et al., 2016). In order to facilitate increased appetite, it is also possible that domesticated salmon will have evolved larger oral–pharyngeal phenotypes to consume the volume of feed needed to sustain growth. Such selection‐induced changes to oral–pharyngeal phenotypes through developmental genes have been linked with pleiotropic eye degeneration in other fish species (Yamamoto et al., 2009). Strong artificial selection on developmental genes controlling oral–pharyngeal phenotypes could therefore also be contributing to the reduction in eye size that we document here and provides an interesting avenue of further investigation.

Adjusting eye width for fork length may not consider the growth of the fish in a common garden, if their growth rates are significantly different. For instance, under farming conditions, one may be observing an ontogenetic reduction in eye width, because a domesticated fish that grows faster than a wild fish is just further along the developmental timeline than wild fish, as highlighted by Devlin et al. (2012). To address this concern, a supplementary analysis was conducted on fish from the Norwegian experiment, which examined growth rate at the family level, and its influence on eye width (Supplementary material). The results demonstrated that growth could be an important explanatory variable in the freshwater life stage, but length is a better predictor of eye width than growth rate in the saltwater life stage, and therefore, trends in adjusted eye width are likely to be due to genetic background, rather than growth dependant ontogenetic changes. In addition, for the naturally reared Irish fish in the freshwater life stage, where developmental stage is most similar between domesticated and wild fish, there was still a significant effect of genetic background on eye width, albeit with no significant pairwise differences between wild and domesticated fish. These results corroborate the trends seen in the artificially reared experiments.

### 4.2 Aquaculture environment and eye size

Reduced adjusted eye size has also been documented in wild Atlantic cod (Wringe et al., 2015) reared under aquaculture conditions, albeit one generation, suggesting that smaller eyes appear to be beneficial, or at no cost to fitness, in the aquaculture environment. Therefore, it is possible that the aquaculture environment could inadvertently select for individuals with smaller eyes in the ~13–15 generations of Atlantic salmon domestication, occurring indirectly, either as a consequence of selection for some other associated trait, or due to relaxed natural or sexual selection (Perry et al., 2019). Inadvertent selection for smaller eyes could be due to the redundancy for high acuity vision as there is a lack of predation and food is provided readily and without effort, which is then combined with the metabolic costs of eyes in that environment. Finally, reduced visual stimuli caused by a smaller eye could also be beneficial in producing a more docile organism, a trait that is thought to be favoured by artificial selection for improved production efficiency (Rauw et al., 2017). This is also consistent with the observation of a reduction in stress responsiveness in domesticated salmon (Solberg, Skaala, et al., 2013). There is a prevailing view that an increase in eye size equates to better vision (Howell et al., 2021), and indeed, it has been demonstrated that when eye size increases, generally, visual acuity increases (Caves et al., 2017), and morphology and eye function can be linked (Lisney & Hawryshyn, 2010). However, the eye is complex, and therefore, changes in eye size do not always translate into measurable functional shifts (Pankhurst & Montgomery, 1994). Therefore, further work is required to assess how both raw eye size, and eye size relative to body size, may translate to visual acuity in Atlantic salmon.

### 4.3 Artificial light regimes in aquaculture and eye size

In addition to the redundancy of high visual acuity, another characteristic of aquaculture environments that may contribute to the reduction of adjusted eye size during domestication is the use of artificial light regimes. Artificial light regimes are used in a multitude of aquaculture species, with several studies examining the impact of different aspects of artificial light on fish physiology, from aggression in Nile tilapia (*Oreochromis niloticus*) (Carvalho et al., 2013) to plasma cortisol levels in rainbow trout (Karakatsouli et al., 2008). In Atlantic salmon, artificial light is used to suppress early maturation, as pubertal development is linked with reduced growth rate (Horizonte et al., 2018). Salmon parr and pre‐smolts are subjected to constant light as a means of accelerating growth prior to transfer into the marine environment (Stefansson et al., 2007).

Not only do larger eyes increase visual acuity, they also increase the sensitivity of the eye to light (Hall & Ross, 2007). Studies on goldfish (*Carassius auratus*) and zebrafish (*Danio rerio*) show that constant light reduces visual sensitivity (Powers et al., 1988) and acuity (Bilotta, 2000), respectively. Therefore, it is possible that the reduction in adjusted eye size seen in domesticated Atlantic salmon could be an evolutionary response in preventing visual sensitivity and acuity loss. Retinal damage has been documented previously in Atlantic salmon subjected to constant high light intensity regimes (Vera & Migaud, 2009), but the evidence is inconsistent (Migaud et al., 2007). The effect of long‐term multigenerational exposure to artificial light regimes needs further exploration. In addition, although gross eye morphology is an important component of vision, teleost fish rely on a high diversity of photopigments and photoreceptor morphology (Kusmic & Gualtieri, 2000); thus, these will be key components in the future work examining the effect of domestication and aquaculture on vision.

### 4.4 Eye size in the wild

Under conditions in the Srahrevagh River, the natural rearing environment used in this study, fewer significant pairwise differences were seen in eye size between genetic backgrounds, including between wild and domesticated backgrounds, when compared to fish reared under artificial conditions. However, for artificially reared fish, where there is no immediate strong selection from aspects such as predation, or lack of food, reduced adjusted eye size persisted. In the wild, however, vision is an important sensory input for both processing predation risk and thus response (Leduc et al., 2010) and feeding (Fraser & Metcalfe, 1997). It is therefore likely that reduced adjusted eye size is a maladaptive trait eliminated by natural selection in the wild, thus removing any significant trend, in line with results from fitness models (Castellani et al., 2018). The risk of being removed from the population through natural selection is also cumulative over time, which could explain why no pairwise differences were seen between smolts measured after approximately 24 months of life in the river, in comparison with parr sampled after seven months in the river (Figure 3c). There is strong evidence here for inherited morphological change in eye size due to artificial selection in farm environments that has negative fitness consequences for the progeny of domesticated farmed salmon in the wild.

### 4.5 Fork length

Under artificial rearing conditions, the offspring of domesticated fish are larger (fork length) compared with wild fish, with hybrids and backcrosses intermediate in size (Figure 2a,b). The highly heritable and additive effect of domestication on growth in Atlantic salmon has been documented previously, both in relation to body weight in the Norwegian fish used in this experiment (Perry et al., 2020), but also in many other experiments (Fleming et al., 2002; Fleming & Einum, 1997; Glover et al., 2009; Harvey, Solberg, Glover, et al., 2016; Solberg et al., 2016; Solberg, Zhang, et al., 2013; Wolters et al., 2009). There were also significant differences in fork length between wild and domesticated genetic backgrounds found in the freshwater life stage of fish reared in the natural environment, although far smaller differences than those seen under artificial conditions, which is consistent with previous findings (Skaala et al., 2019). There were also no significant differences detected between wild and domesticated genetic backgrounds of naturally reared smolts, possibly caused by energy‐budget plasticity, whereby restricted access to food prevents domesticated salmon from acquiring enough energy to utilize their high genetic growth potential (Glover et al., 2018). Previously, domesticated salmon have been shown to be larger than wild smolts when reared in a natural river environment, both in Norway (Skaala et al., 2012, 2019) and in Ireland (Reed et al., 2015), though moderate relative to the magnitude of the differences observed here in fish under culture, and can vary between age groups, cohorts and food resources in a river setting.

## 5 MANAGEMENT IMPLICATIONS

Introgression of domesticated salmon in wild populations is extensive (Glover et al., 2013; Karlsson et al., 2016; Wringe et al., 2018), and the situation is ongoing (Glover, Wennevik, et al., 2020). Here, we demonstrate here that selection associated with domestication under aquaculture conditions has caused significant changes to body length‐adjusted eye size in Atlantic salmon assessed both in Norwegian and in Irish farm environments. Differences in adjusted eye size and body length are not apparent to the same extent, or at all, between domesticated and wild fish (as parr and of sea migrating smolts) when the progeny are reared under natural river conditions (Figure S2). It is possible that differences in adjusted eye size and body length are not being detected in fish reared under natural conditions due to directional selection against maladaptive domesticated‐induced traits. Understanding the role of domestication on eye size, together with associated fitness consequence in the wild, has important implications for risk assessment of escapees on native wild populations. It also suggests that assessing morphological traits in wild populations to quantify the effect of introgression is ineffective, as the true extent of morphological change is likely to be masked by strong natural selection in the wild.

## CONFLICT OF INTEREST

The authors declare no competing interests.

## Supporting information

Supplementary Material

Supplementary Material

Supplementary Material

## Data Availability

All statistical analyses were carried out in R v. 3.6.2 (R Core Team, 2017), with data and code available in the electronic Supplementary material.
