# Supplementary material for: Domestication‐induced reduction in eye size revealed in multiple common garden experiments: The case of Atlantic salmon (Salmo salar L.)
Source: Evol Appl. 2021 Sep 21;14(9):2319–32. doi: 10.1111/eva.13297 (PMC8477603; doi:10.1111/eva.13297)
Supplement: Supplementary file 1 — Supplementary Material [file EVA-14-2319-s001.docx]

**Supplementary materials**

Supplementary table 1 Output from two linear mixed effect models (one for the Norwegian experiment, and one for the Irish experiment) with log10 transformed fork length as a response variable, using the R package emmeans. Output includes: mean fork length in centimetres (emmean), standard error (SE), degrees of freedom (df), lower confidence limit (lower.CL) and upper confidence limit (upper.CL), broken down by experiment, genetic background and life stage.

| **Experiment** | **lifestage** | **Strain** | **emmean** | **SE** | **df** | **lower.CL** | **upper.CL** |
| --- | --- | --- | --- | --- | --- | --- | --- |
| Irish_Artificial | Freshwater | Wild | 1.095 | 0.00496 | 35.7 | 1.085 | 1.105 |
| Irish_Artificial | Freshwater | HWF | 1.117 | 0.00463 | 25.5 | 1.107 | 1.126 |
| Irish_Artificial | Freshwater | HFF | 1.149 | 0.00475 | 19.6 | 1.139 | 1.159 |
| Irish_Artificial | Freshwater | Domesticated | 1.188 | 0.0048 | 18.6 | 1.178 | 1.198 |
| Irish_Artificial | Saltwater | Wild | 1.28 | 0.00643 | 827.9 | 1.267 | 1.293 |
| Irish_Artificial | Saltwater | HWF | 1.316 | 0.00649 | 652.7 | 1.304 | 1.329 |
| Irish_Artificial | Saltwater | HFF | 1.32 | 0.00604 | 532.7 | 1.308 | 1.332 |
| Irish_Artificial | Saltwater | Domesticated | 1.371 | 0.00559 | 532 | 1.36 | 1.382 |
| Irish_Natural | Freshwater | Wild | 0.691 | 0.00566 | 18.1 | 0.679 | 0.703 |
| Irish_Natural | Freshwater | HWF | 0.727 | 0.00526 | 13.5 | 0.716 | 0.739 |
| Irish_Natural | Freshwater | HFF | 0.747 | 0.00494 | 10.5 | 0.736 | 0.758 |
| Irish_Natural | Freshwater | Domesticated | 0.755 | 0.00494 | 10.4 | 0.744 | 0.766 |
| Irish_Natural | Saltwater | Wild | 1.095 | 0.00642 | 352.4 | 1.082 | 1.107 |
| Irish_Natural | Saltwater | HWF | nonEst | NA | NA | NA | NA |
| Irish_Natural | Saltwater | HFF | nonEst | NA | NA | NA | NA |
| Irish_Natural | Saltwater | Domesticated | 1.098 | 0.0106 | 1125.8 | 1.077 | 1.119 |
| Norwegian_Artificial | Freshwater | Wild | 1.19 | 0.0125 | 20.9 | 1.17 | 1.22 |
| Norwegian_Artificial | Freshwater | Wild.BC | 1.23 | 0.0125 | 21.1 | 1.21 | 1.26 |
| Norwegian_Artificial | Freshwater | HWF | 1.29 | 0.0163 | 30.3 | 1.25 | 1.32 |
| Norwegian_Artificial | Freshwater | HFF | 1.29 | 0.0163 | 30.6 | 1.26 | 1.32 |
| Norwegian_Artificial | Freshwater | F2 | 1.28 | 0.0125 | 21.2 | 1.26 | 1.31 |
| Norwegian_Artificial | Freshwater | Domesticated.BC | 1.31 | 0.0125 | 21.4 | 1.29 | 1.34 |
| Norwegian_Artificial | Freshwater | Domesticated | 1.37 | 0.0125 | 21.1 | 1.34 | 1.4 |
| Norwegian_Artificial | Saltwater | Wild | 1.52 | 0.0133 | 25.6 | 1.49 | 1.55 |
| Norwegian_Artificial | Saltwater | Wild.BC | 1.58 | 0.0131 | 24.2 | 1.56 | 1.61 |
| Norwegian_Artificial | Saltwater | HWF | 1.62 | 0.0172 | 36.6 | 1.58 | 1.65 |
| Norwegian_Artificial | Saltwater | HFF | 1.65 | 0.0169 | 34 | 1.62 | 1.69 |
| Norwegian_Artificial | Saltwater | F2 | 1.66 | 0.0132 | 24.4 | 1.63 | 1.68 |
| Norwegian_Artificial | Saltwater | Domesticated.BC | 1.68 | 0.0132 | 24.3 | 1.65 | 1.71 |
| Norwegian_Artificial | Saltwater | Domesticated | 1.71 | 0.0132 | 24.7 | 1.69 | 1.74 |

**
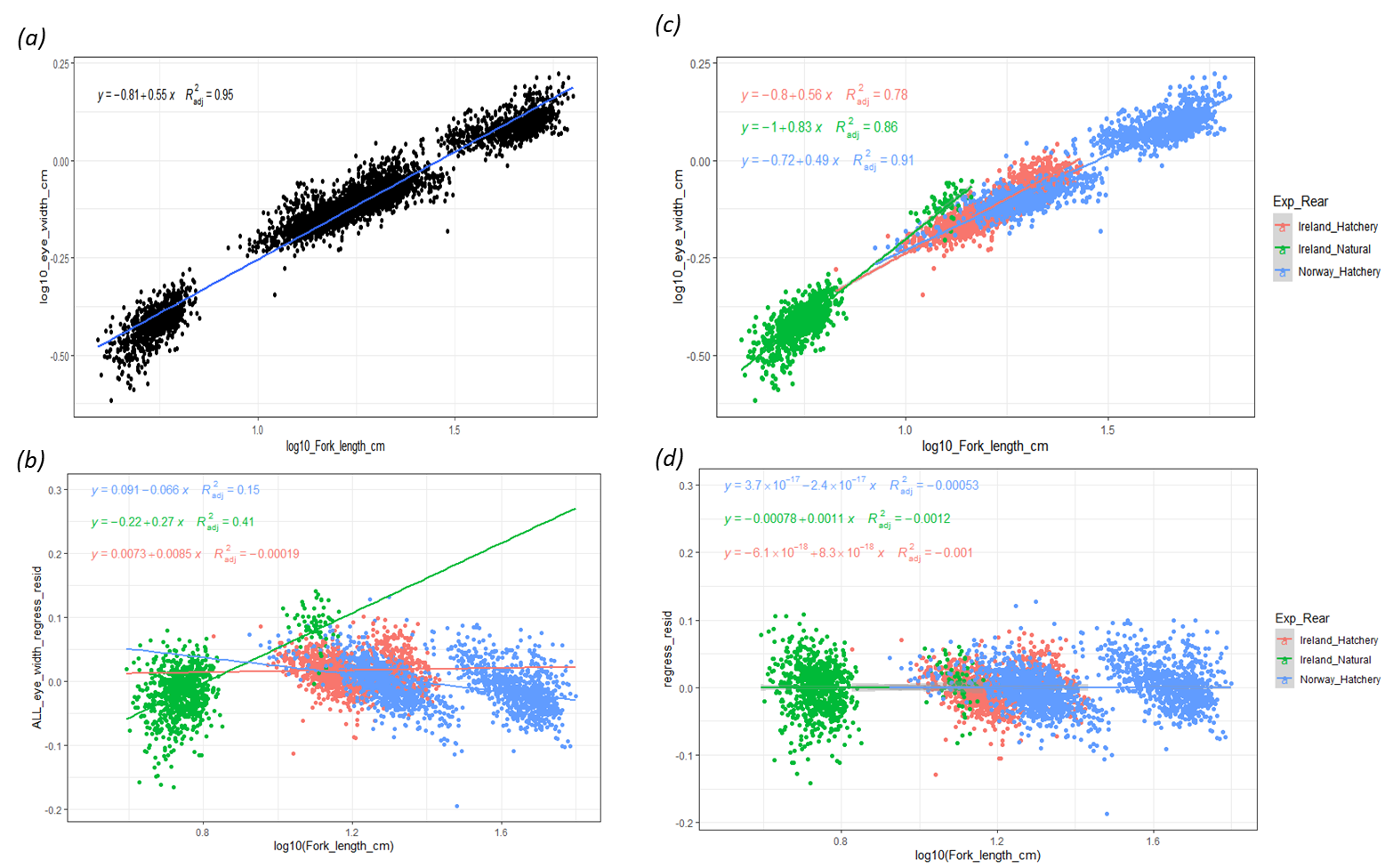
**

**Supplementary figure 1 The difference in allometry between artificially reared and naturally reared fish meant that the residuals from (a) using the residuals from one regression that included all datapoints (not partitioned) (b) did not remove the effect of fork length on characters. Based on this, (c) data was partitioned into three groups: Norway artificially reared, Ireland artificially reared and Ireland naturally reared. By using the (d) residuals from these three separate regressions, the effect of fork length between the three experimental groups was removed. The differences in allometry shown in (c) was further demonstrated by different gradients between the three experimental groups. A linear model exploring the interaction between experimental group and log fork length also demonstrated significant differences (F_2,3709_ = 562.35, Sum Sq = 1.07, p < 0.01).**

**Parentage analysis – Norwegian fish**

Genomic DNA was extracted from alcohol-preserved fin-clip samples of parents and offspring using the Qiagen DNeasy®96 Blood & Tissue Kit, followed by a multiplex PCR which amplified five microsatellite loci; SsaF43 [GenBank:U37494] (Sánchez et al., 1996), Ssa197 [GenBank:U43694.1] (O’Reilly et al., 1996), SSsp3016[GenBank:AY372820], MHCI (Grimholt et al., 2002), MHCII (Grimholt et al., 2002). An ABI Applied Biosystems ABI 3730 Genetic Analyser was used for fragment analysis, the outputs of which were used to call genotypes in GeneMapper (Applied Biosystems, v. 4.0). Offspring were assigned to family by the use of an exclusion based parental assignment program (Family Analysis Program v3.6) (Taggart, 2006). Further details are outlined by (Solberg et al., 2013).

**Parentage analysis – Irish fish**

Genomic DNA was extracted from alcohol-preserved fin-clip samples using the Promega Wizard® SV 96 Genomic DNA Purification System. DNA quality was assessed on agarose gels by comparison with a Quick-Load® Purple 100 bp DNA Ladder (New England Biolabs) and concentration was estimated using a Nanodrop microvolume spectrophotometer (Thermo Fisher Scientific). Aliquots of DNA were diluted to approximately 2-10ng/μL for microsatellite locus amplification. Ten microsatellite DNA loci were amplified in three multiplex panels (Panel 1, Ssa197 (O’Reilly et al., 1996) and MHC2 (Stet et al., 2002); Panel 2, Ssa202, Ssa171 (both O’Reilly et al., 1996), Sssp2210 (Paterson et al. 2004) and SsaD170 (unpublished; EMBL Accession no. AF525205); Panel 3, Ssp2216, Ssp1605 (both Paterson et al., 2004); SsoSL85 (Slettan et al., 1995) and SsaD157 (King et al., 2005)). All PCRs were performed in a total volume of 3.5μL, including 1μL of genomic DNA and 1.75μL Plain Combi PP Master Mix (TopBio). Primer concentrations (same for forward and reverse primers for each locus) and fluorescent label employed in each panel were as follows: Ssa197(VIC) 0.02μM, MHC2(NED) 0.04μM, Ssa202(FAM) 0.06μM, Sssp2210(VIC) 0.03μM, SsaD170(NED) 0.06μM, Ssa171(PET) 0.06μM, Ssp2216(VIC) 0.02μM, SsoSL85(NED) 0.04μM, SsaD157(NED) 0.12μM and Sssp1605(PET) 0.06μM. Forward primers included fluorescent labels from the Applied Biosystems (ABI) standard dye sets to enable visualisation on ABI genetic analysers and reverse primers included a GTTT ‘pig-tail’ to minimise stuttering. Primers to amplify a locus for sex determination was included in Panel 1, SalmoYF (forward primer labelled with VIC (0.015μM forward and reverse primer concentration)) (Paulo Prodohl, pers.comm.). Cycling conditions included an initial denaturing period of 15 minutes at 95°C followed by: five cycles of 30 seconds at 94°C, 90 seconds at 55°C, 1 minute at 72°C; then 22 cycles of 30 seconds at 94°C, 90 seconds at 57°C and 1 minute at 72°C; and a final incubation at60°C for 30 minutes.

Each sample was diluted in Hi-Di™ Formamide with GeneScan™ 600 LIZ™ Dye Size Standard (ThermoFisher Scientific) as an internal size ladder, comparison with which enabled allele size estimation. Samples were denatured by incubation at 95C for 3 minutes and snap-chilled prior to Electrophoresis performed on an ABI3500xl DNA analyser using POP-7™ Polymer. Alleles and genotype calling for each microsatellite locus in each individual were executed using GeneMarker (SoftGenetics).

Genetic treatment groups for fish were determined with a mixture of parentage analysis and population genetics. Parentage analysis was performed in COLONY v2.0.6.5 (Jones & Wang, 2010), which uses a customisable maximum likelihood method. Parent assignments were only accepted if they had bi-parental *P* ≥ 0.95 in ≥ 4/5 repeat runs of COLONY, and if they corresponded to a known artificial cross. See below for description of COLONY settings. Parental genotypes were partially unavailable for one treatment group and completely unavailable for another, so fish without parentage were assigned to treatment groups using two population genetics methods: discriminant function analysis of principal components (90% of PC variance and one discriminant function) and genotype probability (Mossman & Waser, 1999). Both methods were trained with genotypes of known origin, and genetic group assignments were only accepted if both methods agreed.

We performed five repeat runs of COLONY, each with a different random number seed. Each run featured three runs-within-runs, a feature of COLONY in which a single execution performs the analysis 2+ times with different seeds, and returns the run with the highest likelihood. All runs were of ‘short’ duration and ‘low’ likelihood precision, allowed for the possibility of inbreeding, and allowed the updating of allele frequencies.

Details of all other COLONY parameters and annotated R scripts for making consensus provenance assignments will be available on a data depository after publication.


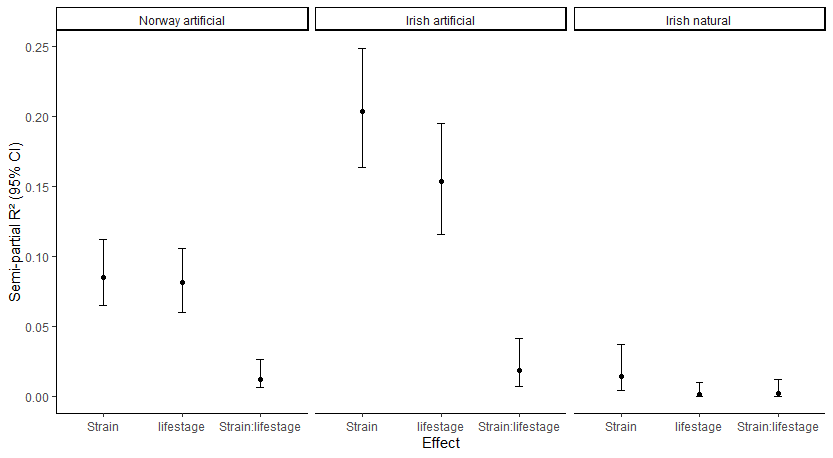


**Supplementary figure 2 Genetic background (here referred to as strain) and life stage both explained a larger proportion of variance in eye size under artificial conditions than under natural conditions.**

**Supplementary growth analyses**

We examined family growth, averaging size of individuals from one family at each life stage (egg diameter, fork length at freshwater, fork length at saltwater). We then compared the growth rate using the following growth equation (Elliott et al., 1995; Solberg et al., 2015):


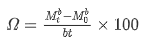


Where M_t_ is the average family fork length at the second measuring time point, M_0_ is the average family fork length at the first measuring time point and b is the species-specific allometric mass exponent describing the relationship between growth rate and body mass. The value for b was set at 0.31, which has been used previously (Elliott et al., 1995; Solberg et al., 2015). Growth could only be assessed in the artificially reared Norwegian experiment, as this was the only experiment that had the necessary family information. Growth rate was calculated between the freshwater and saltwater life stages, which we will refer to as G_1_, and between egg diameter to fork length in the freshwater life stage, which we will refer to as G_0_.

To assess the importance of growth rate and fork length on eye size, two linear regression models were constructed per life stage, with eye width as the response variable and with either growth rate or fork length as the explanatory variable. For eye width in the freshwater life stage, both fork length (R^2^ = 0.85, F_1,34_ = 204.69, p < 0.01) and G_0_ (R^2^ = 0.86, F_1,34_ = 224.69, p < 0.01) had a significant effect, with AICc values of -208.36 and -205.47, respectively. For eye width in the saltwater life stage, both fork length (R^2^ = 0.86, F_1,34_ = 204.69, p < 0.01) and G_1_ (R^2^ = 0.39, F_1,34_ = 211.22, p < 0.01) had a significant effect, with AICc values of -173.04 and -120.88, respectively. Therefore, growth may play an important role in the freshwater life stage, but fork length is a better explanatory variable in the saltwater life stage, as demonstrated by model AICc values. Therefore, the trend we see in the freshwater life stage (domesticated fork length adjusted eye width reduced when compared to wild fork length adjusted eye width) could be due to differences in growth, but this is unlikely in the saltwater life stage.

**References**

Elliott, J. M., Hurley, M. A., & Fryer, R. J. (1995). A New, Improved Growth Model for Brown Trout, Salmo trutta. *Functional Ecology*, *9*(2), 290. https://doi.org/10.2307/2390576

Grimholt, U., Drabløs, F., Jørgensen, S., Høyheim, B., & Stet, R. (2002). The major histocompatibility class I locus in Atlantic salmon ( Salmo salar L.): polymorphism, linkage analysis and protein modelling. *Immunogenetics*, *54*(8), 570–581. https://doi.org/10.1007/s00251-002-0499-8

Jones, O. R., & Wang, J. (2010). COLONY: A program for parentage and sibship inference from multilocus genotype data. *Molecular Ecology Resources*, *10*(3), 551–555. https://doi.org/10.1111/j.1755-0998.2009.02787.x

Mossman, C. A., & Waser, P. M. (1999). Genetic detection of sex-biased dispersal. *Molecular Ecology*, *8*(6), 1063–1067. https://doi.org/10.1046/j.1365-294X.1999.00652.x

O’Reilly, P. T., Hamilton, L. C., McConnell, S. K., & Wright, J. M. (1996). Rapid analysis of genetic variation in Atlantic salmon ( *Salmo salar* ) by PCR multiplexing of dinucleotide and tetranucleotide microsatellites. *Canadian Journal of Fisheries and Aquatic Sciences*, *53*(10), 2292–2298. https://doi.org/10.1139/f96-192

Sánchez, J. A., Clabby, C., Ramos, D., Blanco, G., Flavin, F., Vázquez, E., & Powell, R. (1996). Protein and microsatellite single locus variability in Salmo salar L. (Atlantic salmon). *Heredity*, *77*(4), 423–432. https://doi.org/10.1038/hdy.1996.162

Solberg, M. F., Zhang, Z., & Glover, K. A. (2015). Are farmed salmon more prone to risk than wild salmon? Susceptibility of juvenile farm, hybrid and wild Atlantic salmon Salmo salar L. to an artificial predator. *Applied Animal Behaviour Science*, *162*, 67–80. https://doi.org/10.1016/J.APPLANIM.2014.11.012

Solberg, M. F., Zhang, Z., Nilsen, F., & Glover, K. A. (2013). Growth reaction norms of domesticated, wild and hybrid Atlantic salmon families in response to differing social and physical environments. *BMC Evolutionary Biology*, *13*(1), 234. https://doi.org/10.1186/1471-2148-13-234

Taggart, J. B. (2006). PROGRAM NOTE: FAP: an exclusion-based parental assignment program with enhanced predictive functions. *Molecular Ecology Notes*, *7*(3), 412–415. https://doi.org/10.1111/j.1471-8286.2006.01616.x
